# Sex and Survival After Surgery for Lung Cancer: A Swedish Nationwide Cohort

**DOI:** 10.1016/j.chest.2020.11.010

**Published:** 2020-11-17

**Authors:** Erik Sachs, Ulrik Sartipy, Veronica Jackson

**Affiliations:** a Department of Cardiothoracic Surgery, Karolinska University Hospital, Stockholm, Sweden; b Department of Molecular Medicine and Surgery, Karolinska Institutet, Stockholm, Sweden

**Keywords:** epidemiology (pulmonary), lung cancer, sex, thoracic surgery, ThoR, Swedish National Quality Register for General Thoracic Surgery, VATS, video-assisted thoracic surgery

## Abstract

**Background:**

Prior reports on a possible female survival advantage in both surgical and nonsurgical cohorts of patients with lung cancer are conflicting. Previously reported differences in survival after lung cancer surgery could be the result of insufficient control for disparities in risk factor profiles in men and women.

**Research Question:**

Do women who undergo pulmonary resections for lung cancer have a better prognosis than men when taking a wide range of prognostic factors into account?

**Study Design and Methods:**

We performed a nationwide population-based observational cohort study analyzing sex-specific survival after pulmonary resections for lung cancer. We identified 6356 patients from the Swedish National Quality Register for General Thoracic Surgery and performed individual-level record linkage to other national health-data registers to acquire detailed information regarding comorbidity, socioeconomic status, and vital status. Inverse probability of treatment weighting was used to account for differences in baseline characteristics. The association between female sex and all-cause mortality was assessed with Cox regression models, and flexible parametric survival models were used to estimate the absolute survival differences with 95% CIs. We also estimated the difference in restricted mean survival time.

**Results:**

We observed a lower risk of death in women compared with men (hazard ratio, 0.73; 95% CI, 0.67-0.79). The absolute survival difference at 1, 5, and 10 years was 3.0% (95% CI, 2.2%-3.8%), 10% (95% CI, 7.0%-12%), and 12% (95% CI, 8.5%-15%), respectively. The restricted mean survival time difference at 10 years was 0.84 year (95% CI, 0.61-1.07 years). The findings were consistent across several subgroups.

**Interpretation:**

Women who underwent pulmonary resections for lung cancer had a significantly better prognosis than men. The survival advantage was evident regardless of age, common comorbidities, socioeconomic status, lifestyle factors, physical performance, type and extent of surgery, tumor characteristics, and stage of disease.

**Trial Registry:**

ClinicalTrials.gov; No.: NCT03567538; URL: www.clinicaltrials.gov

FOR EDITORIAL COMMENT, SEE PAGE 1719

Differences in cancer incidence and survival between women and men are known, and the reasons for this are not understood fully.^1^, ^2^, ^3^, ^4^ In Sweden, women have been found to have a lower incidence and less excess mortality than men for most cancers that affect both sexes.^3^ Female sex has been suggested as a risk factor for lung cancer.^5^, ^6^, ^7^ By contrast, several reports exist of a female survival advantage in both surgical and nonsurgical cohorts,^8^, ^9^, ^10^, ^11^, ^12^, ^13^, ^14^ but the results are conflicting.^15^^,^^16^ In a recent Swedish nationwide cohort, women with non-small cell lung cancer consistently were found to have a better prognosis than men.^11^ Diagnostic and treatment intensity was analyzed, and no evidence of differences in clinical management was found. However, the female survival advantage was most pronounced in early-stage lung cancer, that is, patients who were more likely to have undergone surgical treatment. To investigate the association between female sex and better prognosis further, we performed a nationwide population-based study analyzing sex-specific survival after pulmonary resection for lung cancer. We identified the study population from the Swedish National Quality Register for General Thoracic Surgery (ThoR), which contains detailed information on patient characteristics and surgical procedures.

## Methods

In this nationwide population-based observational cohort study, the reporting followed the Strengthening the Reporting of Observational Studies in Epidemiology and the Reporting of Studies Conducted Using Observational Routinely Collected Health Data guidelines for observational studies using routinely collected data.^17^^,^^18^ The study was approved by the Swedish Ethical Review Authority and the need for informed consent was waived (Identifier: 2017/1435-31).

### Study Population

The ThoR register was used to identify the study population.^19^ The ThoR register was started in 2008 and contains detailed information on patient characteristics and surgical procedures for patients who have undergone general thoracic surgery in Sweden. From 2009 through 2011, approximately 50% of all patients who underwent thoracic surgery in Sweden were included. During 2011 and 2012, seven of eight hospitals reported to the register, and complete coverage of all eight thoracic surgery departments in Sweden was achieved in 2013.

### Data Collection

The unique personal identity number that is assigned to all Swedish residents^20^ was used to link information from the ThoR register to other nationwide health-care registers. The record linkage was performed by the Swedish National Board of Health, and the study database subsequently was anonymized. Relevant information on previous medical history was retrieved from the National Patient Register.^21^ Information on educational level, household composition, and household disposable income was obtained from the Longitudinal Integration Database for Insurance and Labor Market Studies.^22^

### Outcomes

The outcome measure was time to death from any cause. The Swedish Population Register was used to ascertain vital status and date of death.^23^

### Definitions

Smoking was divided into four categories: never (never actively smoked), former (smoking cessation more than 1 month before surgery), current (active smoker or smoking cessation within 1 month of surgery), and unknown. Performance status was defined according to the Eastern Cooperative Oncology Group/World Health Organization.^24^ Information on previous or concurrent medical conditions was obtained from the ThoR register or the National Patient Register using International Classification of Diseases codes.^21^ The extent of surgery was classified into two categories: sublobar resection vs lobectomy, bilobectomy, or pneumectomy.

### Statistical Analysis

Baseline characteristics were described with frequencies and percentages for categorical variables and means and SDs for continuous variables. Time to event was calculated as the time in days from the date of surgery to the date of death from any cause or end of follow-up (January 12, 2019). All variables reported in Table 1 were used in the estimation of propensity scores using generalized boosted regression modeling.^25^^,^^26^ We used the scores to develop weights for inverse probability of treatment weighting. We examined the distribution of weights and found no patients with extreme weights, and therefore, we decided that trimming was not necessary. Balance between the groups was assessed by standardized mean differences. An absolute standardized difference of ≤ 0.1 was considered an ideal balance.^27^ All subsequent analyses were conducted in the weighted sample. Cox proportional hazards regression was used to estimate hazard ratios and 95% CIs for the association between female sex and all-cause mortality, using male sex as the reference category. The Cox models was stratified by hospital and year of surgery. We constructed survival curves using the Kaplan-Meier method. We used flexible parametric survival models to obtain survival proportions at specified time points during follow-up together with absolute survival differences with 95% CI.^28^ We estimated the difference in restricted mean survival time in men and women. The restricted mean survival time is a robust measure that represents the mean event-free survival time in a prespecified period.^29^^,^^30^ The statistical analyses were performed with Stata version 16.1 software (StataCorp LP) and included the use of the stpm2^28^ program and R version 3.6.3 software (R Foundation for Statistical Computing) and the twang^26^ package.

**Table 1:** Baseline Characteristics of Patients Who Underwent Pulmonary Resections for Lung Cancer in Sweden From 2008 Through 2017 Before and After IPTW

Variable	Unweighted			IPTW		
	Men	Women	SMD	Men^a^	Women^a^	SMD
No. of patients	2,865	3,671	…	5,991.69	6,164.69	…
Age, y	67.6 ± 9.2	66.8 ± 9.1	0.090	67.1 ± 9.2	67.1 ± 9.1	0.008
BMI, kg/m^2^	26.2 ± 4.3	25.9 ± 5.3	0.070	26.0 ± 4.6	26.0 ± 4.9	0.003
Household composition	…	…	0.174	…	…	0.029
2 adults, no children	1,350 (47.1)	1,482 (40.4)	…	2,629.5 (43.9)	2,673.9 (43.4)	…
1 adult, no children	1,019 (35.6)	1,615 (44.0)	…	2,359.0 (39.4)	2,505.4 (40.6)	…
1-2 adults and ≥1 child(ren)	496 (17.3)	574 (15.6)	…	1,003.2 (16.7)	985.3 (16.0)	…
Education, y	…	…	0.135	…	…	0.021
< 10	1,089 (38.0)	1,162 (31.7)	…	2,109.8 (35.2)	2,110.3 (34.2)	…
10-12	1,222 (42.7)	1,700 (46.3)	…	2,644.1 (44.1)	2,773.7 (45.0)	…
> 12	554 (19.3)	809 (22.0)	…	1,237.8 (20.7)	1,280.7 (20.8)	…
Household disposable income, kSEK	343 ± 347	327 ± 405	0.040	333 ± 296	331 ± 386	0.006
Smoking status	…	…	0.214	…	…	0.042
Never smoker	409 (14.3)	814 (22.2)	…	1,079.4 (18.0)	1,198.6 (19.4)	…
Former smoker	1,586 (55.4)	1,774 (48.3)	…	3,084.0 (51.5)	3,134.4 (50.8)	…
Current smoker	818 (28.6)	1,001 (27.3)	…	1,717.9 (28.7)	1,705.8 (27.7)	…
Unknown	52 (1.8)	82 (2.2)	…	110.4 (1.8)	125.8 (2.0)	…
Alcohol dependency	228 (8.0)	132 (3.6)	0.188	348.4 (5.8)	298.5 (4.8)	0.043
Preoperative predicted FEV_1_ < 80%	1,040 (41.4)	1,195 (36.5)	0.101	2,052.0 (38.8)	2,088.1 (38.1)	0.013
Performance status	…	…	0.151	…	…	0.027
0	1,643 (57.5)	2,357 (64.3)	…	3,627.0 (60.5)	3,803.7 (61.7)	…
1	1,131 (39.6)	1,247 (34.0)	…	2,215.6 (37.0)	2,223.0 (36.1)	…
2+	83 (2.9)	61 (1.7)	…	149.0 (2.5)	137.9 (2.2)	…
Hypertension	1,116 (39.0)	1,225 (33.4)	0.116	2,148.0 (35.9)	2,230.1 (36.2)	0.007
Ischemic heart disease	675 (23.6)	408 (11.1)	0.333	1,032.2 (17.2)	944.4 (15.3)	0.052
Atrial fibrillation	329 (11.5)	210 (5.7)	0.207	531.1 (8.9)	476.6 (7.7)	0.041
Hyperlipidemia	430 (15.0)	372 (10.1)	0.147	746.4 (12.5)	725.6 (11.8)	0.021
Heart failure	210 (7.3)	128 (3.5)	0.171	324.3 (5.4)	293.9 (4.8)	0.029
COPD	458 (16.0)	648 (17.7)	0.045	964.6 (16.1)	1,046.6 (17.0)	0.024
Diabetes mellitus	507 (17.7)	374 (10.2)	0.218	852.8 (14.2)	770.6 (12.5)	0.051
Prior stroke/TIA	305 (10.6)	252 (6.9)	0.134	535.6 (8.9)	492.4 (8.0)	0.034
Peripheral vascular disease	315 (11.0)	169 (4.6)	0.240	477.9 (8.0)	408.5 (6.6)	0.052
Chronic kidney disease	70 (2.4)	59 (1.6)	0.059	116.0 (1.9)	133.9 (2.2)	0.017
Preoperative radiotherapy	95 (3.4)	90 (2.5)	0.053	170.6 (2.9)	153.2 (2.6)	0.023
Preoperative chemotherapy	133 (4.8)	140 (3.9)	0.043	263.0 (4.5)	259.1 (4.3)	0.009
Preoperative PET scanning	2,353 (88.9)	3,007 (88.1)	0.024	4,895.4 (88.4)	5,055.9 (88.5)	0.004
Lobectomy/pneumonectomy	2,268 (79.2)	2,803 (76.4)	0.068	4,712.3 (78.6)	4,777.4 (77.5)	0.028
VATS	510 (17.8)	907 (24.7)	0.169	1,219.3 (20.3)	1,360.3 (22.1)	0.042
Extended resection^b^	135 (4.7)	126 (3.4)	0.065	264.5 (4.4)	241.3 (3.9)	0.025
Lymph node sampling	2,329 (84.2)	2,926 (82.2)	0.052	4,828.7 (83.1)	4,950.2 (82.9)	0.006
Microscopic residual disease	173 (6.0)	169 (4.6)	0.064	322.3 (5.4)	302.8 (4.9)	0.021
Postoperative histopathologic findings	…	…	0.316	…	…	0.062
Squamous cell carcinoma	638 (22.3)	457 (12.4)	…	1053.5 (17.6)	976.6 (15.8)	…
Adenocarcinoma	1,441 (50.3)	2,087 (56.9)	…	3,185.5 (53.2)	3,364.7 (54.6)	…
Carcinoid	154 (5.4)	383 (10.4)	…	455.5 (7.6)	533.0 (8.6)	…
Other	335 (11.7)	390 (10.6)	…	695.4 (11.6)	679.9 (11.0)	…
Unknown	297 (10.4)	354 (9.6)	…	601.8 (10.0)	610.5 (9.9)	…
Stage of disease^c^	…	…	0.206	…	…	0.042
IA	966 (33.7)	1595 (43.4)	…	2,271.0 (37.9)	2,437.8 (39.5)	…
IB	655 (22.9)	705 (19.2)	…	1,291.2 (21.5)	1,281.7 (20.8)	…
IIA	366 (12.8)	396 (10.8)	…	702.2 (11.7)	722.8 (11.7)	…
IIB	319 (11.1)	315 (8.6)	…	611.0 (10.2)	575.5 (9.3)	…
IIIA	377 (13.2)	440 (12.0)	…	755.3 (12.6)	780.2 (12.7)	…
IIIB+	182 (6.4)	220 (6.0)	…	361.1 (6.0)	366.5 (5.9)	…

Data are presented as No. (%) or mean ± SD, unless otherwise indicated. IPTW = inverse probability of treatment weighting; kSEK = 1,000 Swedish Krona; SMD = standardized mean difference; TIA = transient ischemic attack; VATS = video-assisted thoracic surgery.

a The overall numbers of patients in each group are not necessarily integers owing to inverse probability of treatment weighting.

b If any structure other than the lung or lymph nodes was included in the resection (eg, pericardium, diaphragm, thoracic wall).

c Pathologic stage.

### Missing Data

Data were complete for most variables, including exposure and outcome, but the following variables had missing data: preoperative predicted FEV_1_ (11.4%), preoperative PET scanning (7.3%), BMI (6.2%), lymph node sampling (3.2%), preoperative radiotherapy (2.7%), and preoperative chemotherapy (2.7%). For variables with missing data, the weights were constructed also to balance rates of missingness in both groups.^25^^,^^26^

## Results

The study population consisted of 6,536 patients (56% women, 44% men) who underwent pulmonary resection for lung cancer in Sweden from 2008 through 2017. The mean age was 67 years for women and 68 years for men. Women were more likely never to have smoked and less likely to have comorbidities (including alcohol dependency), with the exception of COPD. At the time of surgery, women were found to have a better functional status and greater pulmonary function than men, and women underwent minimally invasive and sublobar resections more often than men. Adenocarcinoma was more common in women and squamous cell carcinoma was found more often in men. Compared with men, women had a higher educational level, a lower income, and more often lived alone. Baseline characteristics according to sex before and after inverse probability of treatment weighting are presented in Table 1. The distribution of baseline characteristics was well balanced after inverse probability of treatment weighting, and the standardized mean difference was < 0.1 in all variables (Table 1, e-Fig 1).

### Survival

**Figure 1:**
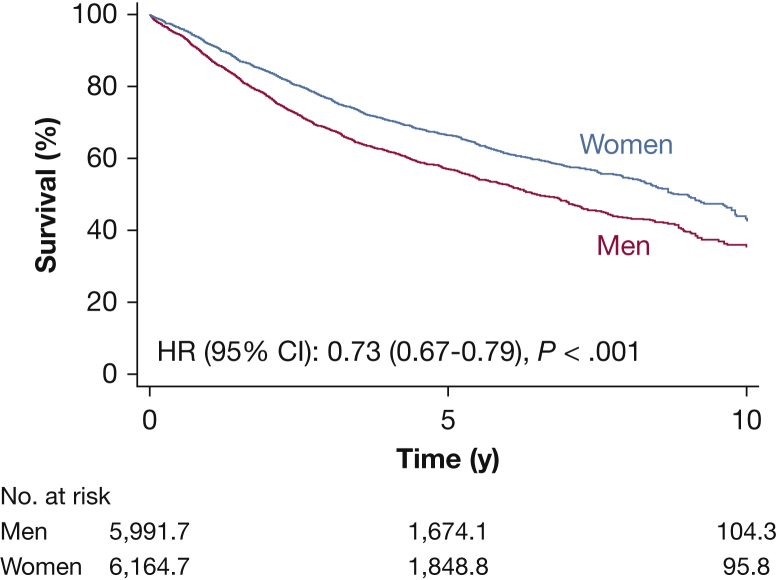
Kaplan-Meier estimated survival curve plotted against time from surgery and stratified according to sex. Male patients are the reference group. The numbers of patients at risk are not necessarily integers owing to inverse probability of treatment weighting. HR = hazard ratio.

Women consistently were found to achieve better survival than men, and the survival gap increased over the years (Fig 1, Table 2). We observed a lower risk of death in women compared with men (hazard ratio, 0.73; 95% CI, 0.67-0.79). The absolute survival difference at 1, 5, and 10 years was 3.0% (95% CI, 2.2%-3.8%), 10% (95% CI, 7.0%-12%), and 12% (95% CI, 8.5%-15%), respectively. The restricted mean survival time at 10 years was 6.8 years (95% CI, 6.7-7.0 years) and 6.0 years (95% CI, 5.8-6.2 years) for women and men, respectively. The restricted mean survival time difference at 10 years was 0.84 year (95% CI, 0.61-1.07 years) (e-Fig 2). The overall survival was fairly stable over the study period, and the sex-specific 3-year survival according to year of surgery showed a consistent pattern of better survival for women compared with men (e-Fig 3). Early mortality, defined as death within 30 days of surgery, was 1.4% in men vs 0.7% in women (*P* = .010).

**Table 2:** Survival According to Sex in Patients Who Underwent Pulmonary Resection for Lung Cancer in Sweden From 2008 Through 2017^a^

Time From Surgery, y	Survival		Survival Difference
	Men	Women	
1	89 (88-90)	92 (91-92)	3.0 (2.2-3.8)
5	56 (54-58)	66 (64-68)	10 (7.0-12)
10	36 (33-39)	48 (45-50)	12 (8.5-15)

Data are presented as percentage (95% CI).

a After inverse probability of treatment weighting.

### Age, Histopathologic Findings, and Stage

**Figure 2:**
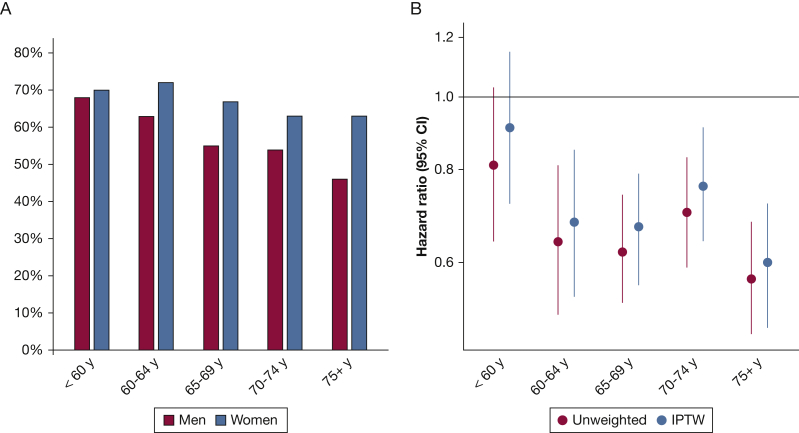
A, Bar graph showing survival at 5 years in age categories according to sex. B, Hazard ratios and 95% CIs for the association between female sex and all-cause mortality in the unweighted (red) and weighted (blue) population in different age categories. Male patients are the reference group. IPTW = inverse probability of treatment weighting.

**Figure 3:**
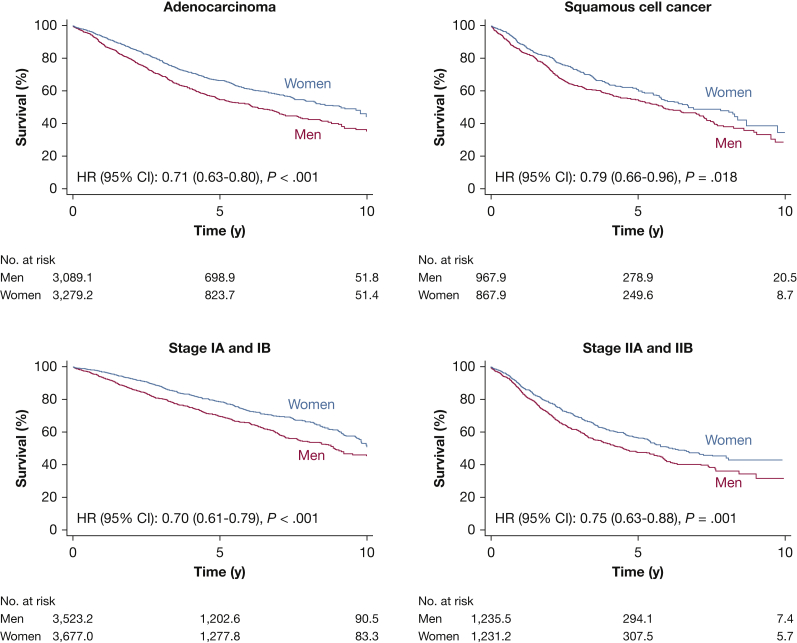
Kaplan-Meier estimated survival curves plotted against time from surgery and stratified according to sex in different subsets of patients. A, Adenocarcinoma. B, Squamous cell carcinoma. C, Stage IA and IB disease. D, Stage IIA and IIB disease. The numbers of patients at risk are not necessarily integers owing to inverse probability of treatment weighting. HR = hazard ratio.

Separate analyses in subsets of patients according to age categories, histopathologic findings, and pathologic stage revealed the same pattern, with few exceptions. Women showed a better 5-year survival compared with men in all age categories, except in patients younger than 60 years (Fig 2, Table 3). Figure 3 shows that the female survival advantage was seen in patients with adenocarcinoma as well as in patients with squamous cell carcinoma, although the survival difference was less pronounced in patients with squamous cell carcinoma. Women with stage I-II disease showed better survival than men with an approximately 9% absolute survival difference at 5 years in both stages (Fig 3, Table 3).

**Table 3:** Survival According to Sex in Different Subsets of Patients Who Underwent Pulmonary Resection for Lung Cancer in Sweden From 2008 Through 2017^a^

Variable	5-y Survival		Survival Difference
	Men	Women	
Age categories, y			
<60	68 (64-73)	70 (66-74)	1.7 (−4.4 to 7.8)
60-64	63 (58-68)	72 (68-76)	9.4 (3.2-16)
65-69	55 (51-59)	67 (64-71)	12 (7.2-17)
70-74	54 (50-58)	63 (59-67)	8.9 (3.3-14)
75+	46 (42-52)	63 (59-67)	16 (10-23)
Histopathologic findings			
Squamous cell carcinoma	52 (48-56)	59 (55-64)	7.3 (1.1-13)
Adenocarcinoma	55 (52-58)	66 (64-69)	11 (7.3-15)
Stage of disease			
IA and IB	70 (67-72)	78 (76-80)	8.6 (5.6-12)
IIA and IIB	47 (43-51)	56 (52-60)	9.1 (3.5-15)

Data are presented as percentage (95% CI).

a After inverse probability of treatment weighting.

## Discussion

In this nationwide population-based study of patients undergoing pulmonary resection for lung cancer, women consistently were found to have a better prognosis than men. The female survival advantage was independent of differences in baseline characteristics such as comorbidities, physical frailty, socioeconomic status, and tumor characteristics.

The female survival advantage was evident regardless of age, except in patients who were 60 years or younger, which has been reported previously.^31^ It was suggested that the lack of an evident survival difference in the younger age category may be explained by differences in life expectancy between women and men. By contrast, a Norwegian study, analyzing sex-specific long-term survival after lung cancer surgery and taking median expected lifetime into account, found that female sex was associated independently with better outcome.^8^ The reasons for the lack of a female survival advantage in the younger age category in our cohort remain uncertain. Factors such as more aggressive treatment regimens in younger patients and lifestyle choices, before and after an early diagnosis of lung cancer, may influence survival.^32^^,^^33^

Life expectancy is affected by smoking patterns in the general population, and tobacco smoking is one of the most important risk factors of disease burden and mortality.^34^, ^35^, ^36^ In the present cohort, women more often were never smokers, whereas men were more likely to be former smokers, which is in accord with previous reports.^11^^,^^15^^,^^37^ An evaluation of lung cancer risk in young Swedish women found first-hand and second-hand exposure to tobacco smoke to be the greatest risk factor also for nonsmokers when taking other risk factors such as lifestyle, environmental exposures, and personal and family medical history into account.^38^ A shorter lung cancer latency in younger women, compared with older women, also was reported. Since the 1980s, overall daily tobacco smoking incidence has decreased in Sweden, and in 2018, 7% of the Swedish population reported daily tobacco smoking, with no evident difference between the sexes.^34^ However, smoking rates have been higher in women than in men from the early 1990s. The gradual decline in smoking prevalence has been steeper in men than in women, especially among younger individuals. In a prospective evaluation of lung cancer risk and survival, women were found to have a prevalence OR of 1.9 (95% CI, 1.5-2.5) and a hazard ratio of fatal outcome of 0.48 (95% CI, 0.25-0.89) compared with men of equal age and exposure to tobacco smoke.^7^ The authors concluded that women might have an increased susceptibility to tobacco carcinogens, a notion supported by some^39^ and contradicted by others.^40^ Thus, differences in smoking prevalence, disease latency, and susceptibility to the harmful effects of tobacco smoke may be contributing explanatory factors for the lack of a female survival advantage in the younger patients.

A comparison of the contribution of smoking-related deaths to differences in life expectancy among the Nordic countries, using lung cancer mortality as proxy, has been conducted.^41^ The largest differences in life expectancy were seen between Denmark and Sweden and the smallest were between Norway and Sweden. Men had a 0.39-year shorter life expectancy (0.25 year [64%] attributable to smoking) and women had a 0.06-year shorter life expectancy (0.07 year [116%] attributable to smoking) in Norway compared with Sweden.

The average length of life in Sweden has increased more in men than in women, and from 1980 to 2019, the survival gap diminished from 6.1 to 3.4 years.^42^ Using lung cancer mortality as a proxy, the contribution of smoking-related deaths to the narrowing survival gap was estimated to be 0.6 year (38%) from 1997 through 2016.^43^ Thus, the survival gap diminished by 2.7 years in four decades,^42^ which is roughly a decrease of 0.68 year per decade. The contribution of smoking-related deaths to the narrowing survival gap could be estimated to be approximately 0.3 year per decade. Together with the finding that the female survival advantage is independent of life expectancy in Norway,^8^ and considering the small differences in life expectancy between Sweden and Norway,^41^ our finding of a restricted mean survival time difference at 10 years of 0.84 year (95% CI, 0.61-1.07 years) in favor of women may suggest a female survival advantage in patients undergoing surgery for lung cancer in Sweden that cannot be explained fully by differences in life expectancy and smoking patterns in the general population.

Socioeconomic disparities have been shown to influence lung cancer survival.^44^, ^45^, ^46^ Women in this cohort achieved a higher educational level than men, which has been linked to a better prognosis.^46^ However, women had lower incomes and more often lived alone, factors that have been associated with poorer survival.^44^^,^^45^ Socioeconomic status also has been linked to lifestyle behaviors such as alcohol consumption.^47^^,^^48^ In Sweden, excessive alcohol consumption has been found to be higher among those with an intermediate level of education as compared with those with the highest or the lowest educational levels.^47^ From 2006 through 2018, the prevalence of excessive alcohol consumption was higher in men than in women (20% vs 13%) when taking age, educational level, region, and country of birth into account. In the current cohort, men showed a higher prevalence of alcohol dependency than women. Moderate alcohol consumption has been associated with a modest decrease in lung cancer risk, whereas high alcohol consumption has been associated with an increased risk of lung cancer and lung adenocarcinoma.^49^ By contrast, squamous cell carcinoma was associated inversely with any level of alcohol drinking. Alcohol dependency also has been associated with an increased risk of postoperative complications in patients undergoing lung cancer surgery^50^^,^^51^; however, the results are conflicting regarding the effect on mortality.^50^, ^51^, ^52^, ^53^ Taken together, efforts aiming to reduce excessive alcohol consumption and to prevent dependency may have the potential to decrease morbidity and mortality in patients with lung cancer, particularly among men.

Cardiovascular disease has been associated with increased lung cancer risk and mortality.^54^, ^55^, ^56^, ^57^ By contrast, dyslipidemia has been associated with a lower risk of lung cancer,^58^ and the use of statins has been associated with reduced lung cancer risk and mortality.^59^^,^^60^ In the present cohort, both manifest cardiovascular disease and risk factors thereof were seen more often in men than in women. Thus, with the exception of a higher prevalence of hyperlipidemia and a slightly lower prevalence of COPD, men in the present cohort showed to a higher extent than women several factors that may affect survival in patients with lung cancer negatively.

TNM stage has been suggested to be the most important prognostic factor in non-small cell lung cancer.^61^^,^^62^ It also has been proposed that TNM staging should be sex specific because men seem to have a poorer prognosis than women within each stage.^14^ Similar to Radkiewicz et al,^11^ we found an absolute survival difference favoring women of 11%, 7.3%, 8.6%, and 9.1% for patients with adenocarcinoma, squamous cell carcinoma, stage I disease, and stage II disease, respectively.

It has been suggested that the female survival advantage may be attributed to factors other than a high prevalence of adenocarcinoma and early-stage disease among women.^63^ Watanabe et al^63^ analyzed postoperative recurrence patterns of non-small cell lung cancer and found that women show delayed times of peak recurrence compared with men. This was evident for stages IB through IIIB and was more pronounced in patients with squamous cell carcinoma. A sex-specific analysis of the risk of stroke after lung cancer diagnosis showed an increased risk of stroke within 1 year after diagnosis for men as compared with 2 years for women.^64^ Delayed postdiagnosis complications and postoperative tumor recurrence in women may have an influence on survival differences in patients with lung cancer.

It has been speculated that survival differences in lung cancer likely are explained by biological differences between women and men.^11^^,^^63^ Radkiewicz et al^11^ found no evidence of unequal treatment between women and men in patients with early-stage lung cancer in Sweden. However, the crude analysis of baseline characteristics in the present cohort support the notion that differences in clinical management exist^31^ and may contribute to sex-specific differences in survival in patients undergoing lung cancer surgery. Preoperative PET scanning, intraoperative lymph node sampling, and lobectomy were more common in men, whereas video-assisted thoracic surgery (VATS) was more common in women.

VATS for early-stage lung cancer has been associated with reduced postoperative pain and complication rates and improved recovery, quality of life, and long-term survival compared with open surgery.^65^, ^66^, ^67^ The adoption of VATS anatomic resections for lung cancer initially was gradual. In recent years, the technique has gained widespread acceptance within the thoracic community.^68^ The adoption of new surgical techniques entails a learning curve,^69^ and therefore, it is plausible that this period also includes patient selection. Hence, it is possible that patients with fewer comorbidities, better functional status, greater pulmonary function, and small tumors (ie, often women) to some extent were more likely to have been selected for VATS resection. Regarding extent of surgery and survival, lobectomy is still considered to be the standard surgical treatment for lung cancer. Anatomic segmentectomy is deemed superior to wedge resections and is considered acceptable for early types of adenocarcinoma.^70^ Women in the present cohort underwent segmentectomies slightly more often than men (4.6% vs 3.2%); however, segmentectomies constituted only 4% of the total number of operations. Thus, women undergoing VATS and segmentectomies to a greater extent than men theoretically could be contributory factors to better prognosis. The slightly less frequent use of preoperative PET scanning and intraoperative lymph node sampling in women could be interpreted as indicative of a greater rate of inadequate staging in women, possibly negatively influencing survival.^70^^,^^71^

Taken together, differences in clinical management of patients with lung cancer may exist, some potentially favoring survival in women and others favoring survival in men. Health-care decisions inadvertently may be influenced by sociocultural conceptions and norms, which might lead to unequal clinical management, as reviewed by Hay et al.^72^ Traditional norms within society influence priorities within health services, and inequalities in health care affect both sexes. For example, perceiving men as strong and in less need of care can lead to a lesser focus on men’s health, despite generally a higher health risk and shorter life expectancy. Norms related to masculinity have been associated with behavioral risks such as substance use and delayed health seeking. Valuing women based on their reproductive capacity in conjunction with the sometimes unrecognized higher risk of health burdens resulting from ageing can lead to worse care of women compared with men. Futures studies prospectively exploring possible sex-specific differences in lifestyle factors, sociocultural conditions, and clinical management of patients with lung cancer are needed. Educational efforts to increase awareness among health-care personnel of inequalities in health care may help to mitigate disparities in clinical management.

Strengths of this study include the nationwide population-based design and the use of national high-quality registers with high coverage and validity, minimizing the risk of selection and misclassification bias. The Swedish National Registers offer detailed prognostic information and complete and accurate follow-up. In addition, the ThoR register contains detailed individual-level information on baseline characteristics, including perioperative parameters. The lack of information on potential confounding factors such as smoking intensity, diet, physical activity, social support, as well as other unknown prognostic factors was an important limitation of our study. However, under the assumption that factors indicating a healthy lifestyle, as well as access to social support, may be more prevalent among women and may affect survival positively, it can be speculated that these limitations would not change the basic conclusions of the present study. Moreover, we did not have information on adjuvant treatment, cause of death, and when and to what extent implementation of VATS and enhanced recovery protocols took place during the study period. These are all factors that might have affected survival; however, because of the high concordance between our results and previous reports on lung cancer-specific survival in the Swedish population, in conjunction with the nationwide population-based study design, we believe our results to be robust.

## Interpretation

Women who underwent pulmonary resection for lung cancer had significantly better prognosis than men. The survival advantage was evident regardless of age, common comorbidities, socioeconomic status, lifestyle factors, physical performance, type and extent of surgery, tumor characteristics, and stage of disease.

## Supplementary Data

e-Online Data
