# Supplementary material for: Sex and Survival After Surgery for Lung Cancer: A Swedish Nationwide Cohort
Source: Chest. 2020 Nov 17;159(5):2029–39. doi: 10.1016/j.chest.2020.11.010 (PMC8129733; doi:10.1016/j.chest.2020.11.010)
Supplement: e-Online Data [file mmc1.pdf]

CHEST Online Supplement

Sex and Survival After Surgery for Lung Cancer

A Swedish Nationwide Cohort

Erik Sachs, MD; Ulrik Sartipy, MD, PhD; and Veronica Jackson, MD, PhD

CHEST 2021; 159(5):2029-2039

Online supplements are not copyedited prior to posting and the author(s) take full responsibility for the accuracy of all data.

© 2021 AMERICAN COLLEGE OF CHEST PHYSICIANS. Reproduction of this article is prohibited without written permission from the American College of Chest Physicians. See online for more details. **DOI:** 10.1016/j.chest.2020.11.010

e-Figure 1. Absolute standardized differences before (hollow circles) and after (filled circles) inverse probability of treatment weighting in the total study population.

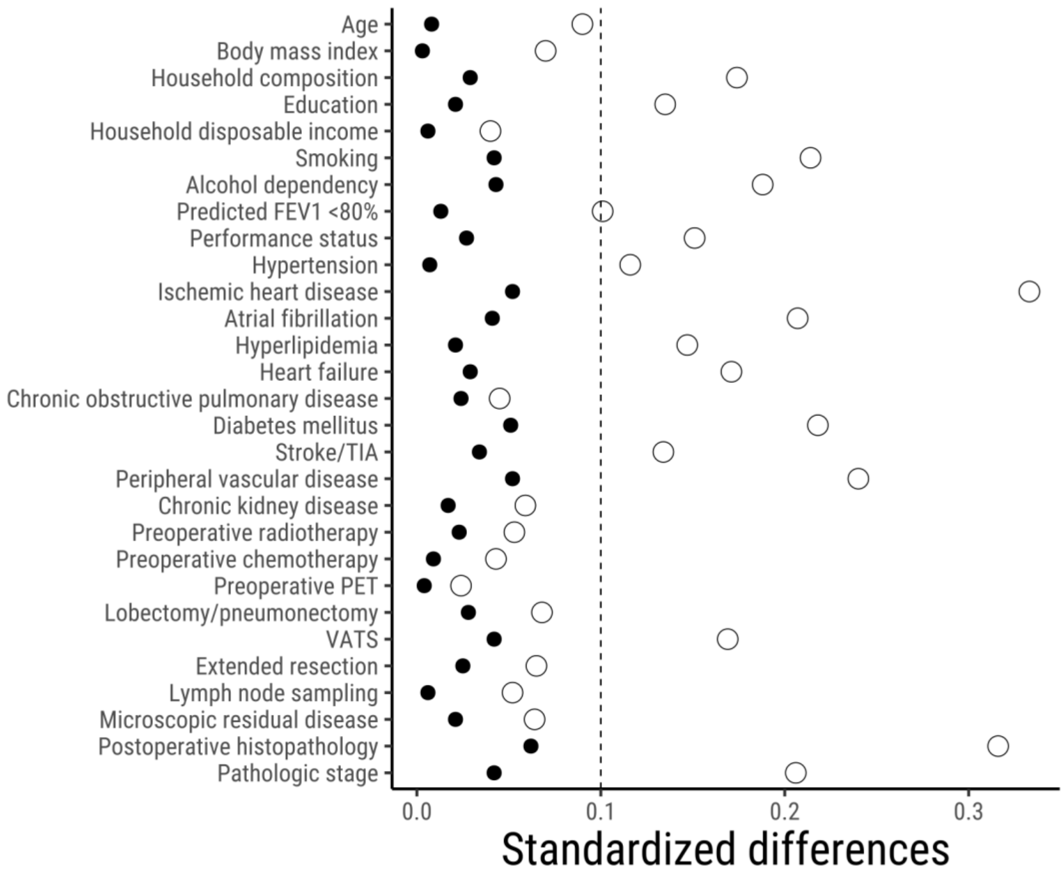

CHEST Online Supplement

e-Figure 2. The difference in restricted mean survival time in years plotted against time from surgery. The shaded area shows the 95% confidence interval. The reference category is men. Women had significantly longer restricted mean survival time than men at all time-points after surgery. *RMST = restricted mean survival time.*

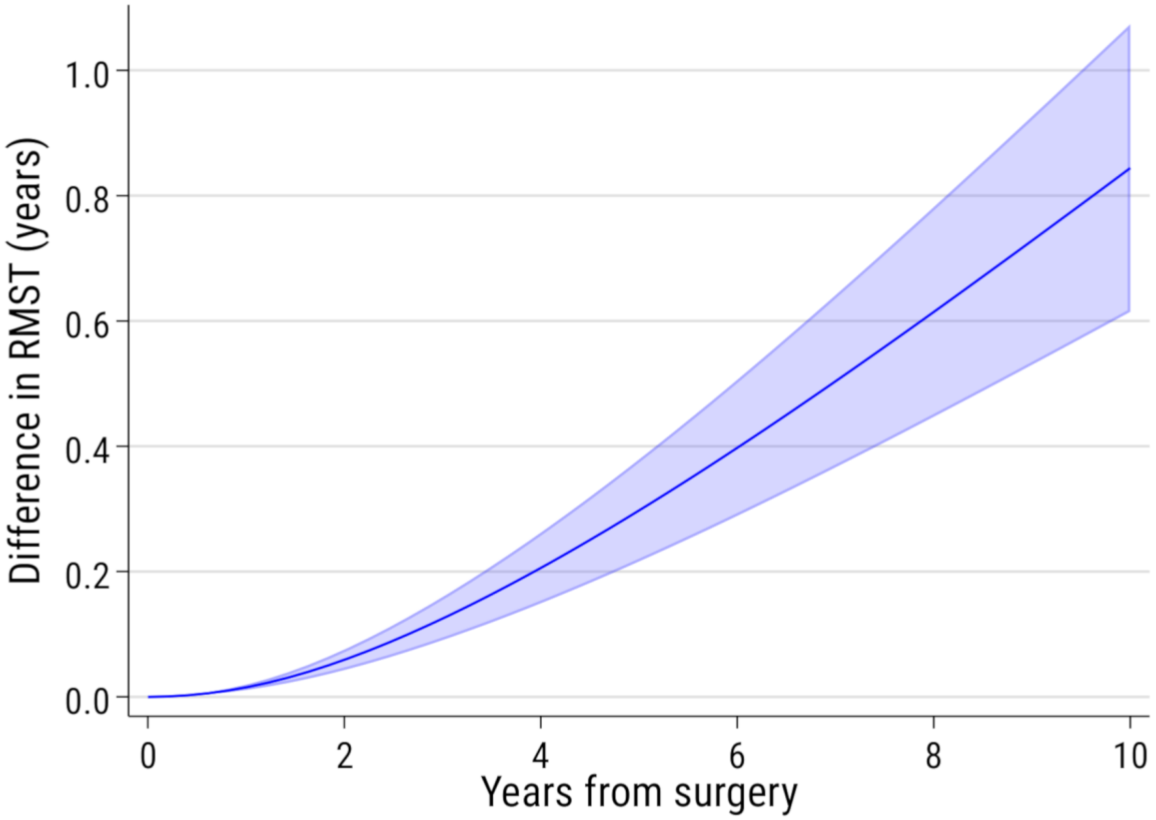

Online supplements are not copyedited prior to posting and the author(s) take full responsibility for the accuracy of all data.

e-Figure 3. Overall 3-year survival according to year of surgery and sex.

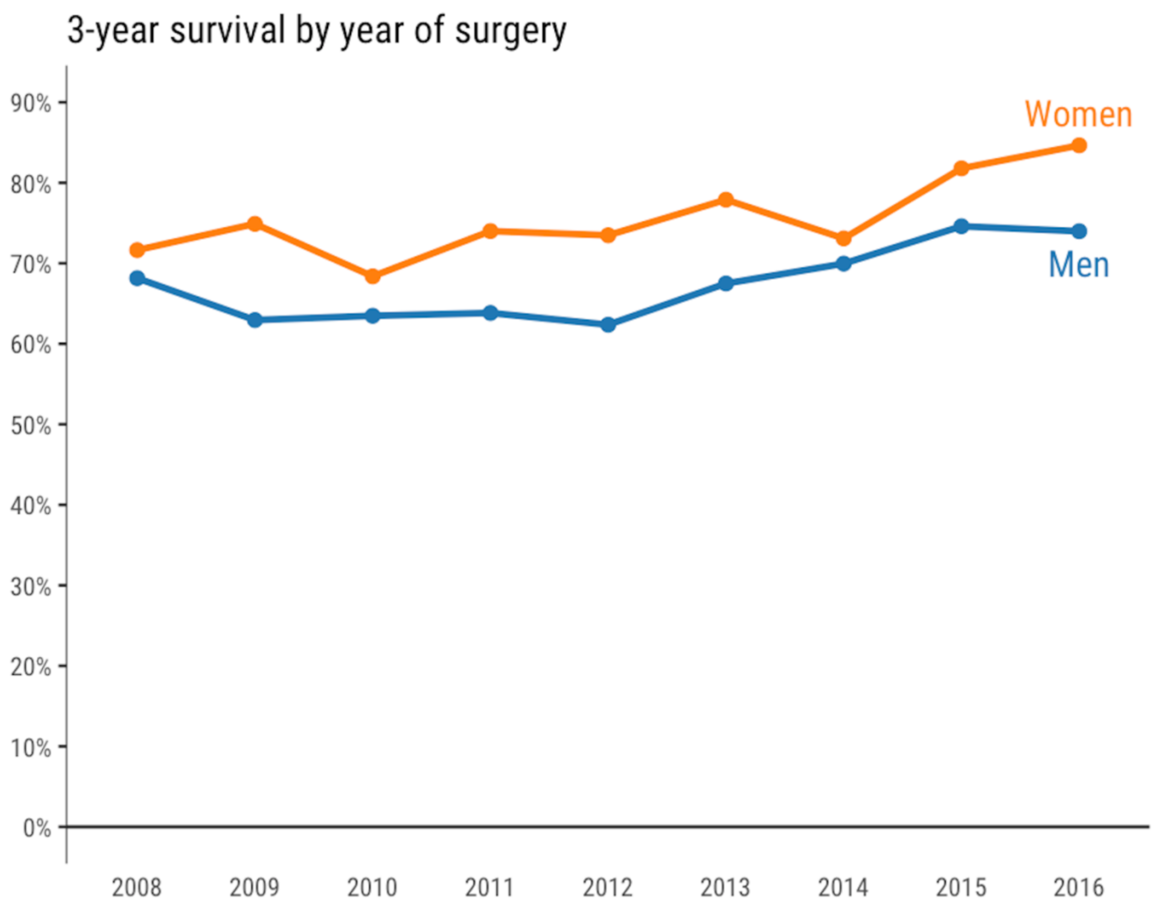

203395

Online supplements are not copyedited prior to posting and the author(s) take full responsibility for the accuracy of all data.
